# Acid Microenvironment in Bone Sarcomas

**DOI:** 10.3390/cancers13153848

**Published:** 2021-07-30

**Authors:** Gemma Di Pompo, Margherita Cortini, Nicola Baldini, Sofia Avnet

**Affiliations:** 1 Biomedical Science and Technologies Lab, IRCCS Istituto Ortopedico Rizzoli, 40136 Bologna, Italy; gemma.dipompo@ior.it (G.D.P.); margherita.cortini@ior.it (M.C.); nicola.baldini@ior.it (N.B.); 2 Department of Biomedical and Neuromotor Sciences, University of Bologna, 40126 Bologna, Italy

**Keywords:** bone sarcoma, extracellular acidosis, tumour microenvironment, tumour-associated stroma, acid-sensing ion channels, vacuolar-ATP-ase, carbonic anhydrase IX, acridine orange

## Abstract

**Simple Summary:**

Although rare, malignant bone sarcomas have devastating clinical implications for the health and survival of young adults and children. To date, efforts to identify the molecular drivers and targets have focused on cancer cells or on the interplay between cancer cells and stromal cells in the tumour microenvironment. On the contrary, in the current literature, the role of the chemical-physical conditions of the tumour microenvironment that may be implicated in sarcoma aggressiveness and progression are poorly reported and discussed. Among these, extracellular acidosis is a well-recognized hallmark of bone sarcomas and promotes cancer growth and dissemination but data presented on this topic are fragmented. Hence, we intended to provide a general and comprehensive overview of the causes and implications of acidosis in bone sarcoma.

**Abstract:**

In bone sarcomas, extracellular proton accumulation is an intrinsic driver of malignancy. Extracellular acidosis increases stemness, invasion, angiogenesis, metastasis, and resistance to therapy of cancer cells. It reprograms tumour-associated stroma into a protumour phenotype through the release of inflammatory cytokines. It affects bone homeostasis, as extracellular proton accumulation is perceived by acid-sensing ion channels located at the cell membrane of normal bone cells. In bone, acidosis results from the altered glycolytic metabolism of bone cancer cells and the resorption activity of tumour-induced osteoclasts that share the same ecosystem. Proton extrusion activity is mediated by extruders and transporters located at the cell membrane of normal and transformed cells, including vacuolar ATPase and carbonic anhydrase IX, or by the release of highly acidic lysosomes by exocytosis. To date, a number of investigations have focused on the effects of acidosis and its inhibition in bone sarcomas, including studies evaluating the use of photodynamic therapy. In this review, we will discuss the current status of all findings on extracellular acidosis in bone sarcomas, with a specific focus on the characteristics of the bone microenvironment and the acid-targeting therapeutic approaches that are currently being evaluated.

## 1. Introduction

### 1.1. Bone Sarcomas

Bone sarcomas comprise highly heterogeneous subtypes of mesenchymal tumours originating from the bone. The most common types of bone sarcoma are osteosarcoma, Ewing’s sarcoma, and chondrosarcoma. Bone sarcomas account for <0.2% of malignant neoplasms registered in the EUROCARE (European Cancer Registry-based study on survival and care of cancer patients) database [1] and their incidence varies according to the different histotype. Osteosarcoma is the first primary cancer of bone (incidence: 0.3 per 100,000 per year), with a higher incidence in adolescents (0.8–1.1 per 100,000 per year at age 15–19 years) [2,3]. Ewing’s sarcoma is the second most common primary malignant bone tumour. It occurs most frequently in children and adolescents, but adults can also be affected. Chondrosarcoma is the most frequent bone sarcoma of adulthood. The incidence is around 0.2 per 100,000 per year, with a median age at diagnosis between 30 and 60 years [2,3]. The survival rate after 5 years for patients with localised primary tumour is 60–70% and 50–60% for osteosarcoma and Ewing’s sarcoma, respectively, with a dramatic drop to 30% for the former and to only around 20% for the latter, in metastatic patients. The survival rate of chondrosarcoma is 50–60% at 10 years according to the histological grade [4]. Current treatments for osteosarcoma and Ewing’s sarcoma combine surgery (preoperative or neoadjuvant), followed by chemotherapy (postoperative or neoadjuvant), and long-term polychemotherapy [5,6]. However, most conventional chemotherapy commonly fails, leading to the cogent need for the identification of novel therapeutic targets and the development of more effective approaches. Among them, the employment of tyrosine kinase and cyclin-dependent kinase inhibitors, DNA repair or chemoresistance targeting, and immunotherapies are currently the most attractive [7].

### 1.2. Cancer-Associated Extracellular Acidosis

Extracellular acidosis is a well-established hallmark of malignancy in solid tumours [8]. Similarly to hypoxia [9,10,11], it influences tumour cell behaviour and clinical outcome by modulating cancer stemness, invasion, invadopodia formation, metastasis, anticancer immune reaction, and response to therapy [8,12].

Solid tumours, including sarcomas [12,13,14], are characterised by an extracellular pH (pHe) that ranges from 6.4 to 7.3, whereas in normal tissues, the range is 7.2 to 7.5 [15]. Tumour formation and progression are strongly influenced by biophysical factors including extracellular acidosis. Understanding how sarcoma cells cope and adapt to the microenvironmental stress that is promoted by an excess of extracellular protons will contribute to a better knowledge of sarcoma pathophysiology and the identification of novel anticancer strategies.

In this review, we will discuss the current status of knowledge on interstitial acidosis in bone sarcomas, taking also into consideration the unique characteristics of sarcoma cells in the bone microenvironment and the acidtargeting therapeutic approaches that are under investigation.

## 2. Source of Acidosis in the Microenvironment of Bone Sarcomas

Acidosis in bone sarcomas is mainly due to (1) the metabolic switch of cancer cells to glycolytic metabolism which, in turn, causes the efflux of lactic acid and protons in the extracellular space; (2) the active release of protons by normal bone cells, mainly osteoclasts, to resorb bone during the formation of osteolytic lesions that occurs with tumour expansion.

### 2.1. Altered Tumour Metabolism and Intratumoural Acidosis in Bone Sarcomas

High glycolytic activity is a common feature of many cancer types, including sarcomas [10,16,17,18,19]. Cancer cells switch to a glycolytic phenotype in a poorly perfused environment. However, as elegantly described by Otto Warburg in 1927 [20,21], glycolysis in cancer cells also occurs under conditions of normal oxygen tension.

In bone cancers, hypoxia results from increased proliferation of cancer cells in association with a high rate of oxygen consumption, and from the intrinsic hypoxia of the bone microenvironment. Indeed, hypoxia greatly influences bone biology and physiology [22]. As a demonstration, in the medullary cavity of animal models, pO_2_ values range from 11.7 to 31.7 mm Hg (1.5–4.2%), with a mean of 20.4 mm Hg (2.7%) [23].

The switch to glycolysis, both under normoxic and hypoxic conditions, follows the activation of hypoxia-inducible factor-1 (HIF-1), which drives the transcription of crucial enzymes of the glycolytic pathway [24]. As an end result, the increased glycolytic rate leads to intracellular accumulation of protons in the cytoplasm, but also the release of protons into the extracellular space as a waste product along with lactate. To survive this harsh microenvironment, cancer cells develop adaptive mechanisms, including transcriptional, posttranslational, and morphological alterations, which eventually lead to profound changes in their phenotype and the metabolic profile [12]. Cancer-associated acidosis is attracting increasing interest in the field of cancer research. New in vivo imaging tools are being developed to assess the association between cancer metabolism and the acidic microenvironment [25]. For example, in a near future, it will be possible to combine ^8^F-FDG PET, currently used for staging bone sarcomas [26,27], together with chemical exchange saturation transfer magnetic resonance imaging (CEST-MRI), to detect acidic regions of the tumour in order to determine its metastatic potential.

Finally, an additional metabolic trigger of tumour interstitial acidosis, in addition to glycolysis exacerbation, could be the hydration of excessive CO_2_ production in the more oxidative areas of the tumour [28]. However, this mechanism has not been explored in bone sarcoma.

In this context, although not thoroughly explored, it is noteworthy that acidosis, conversely, may lead to HIF-1 regulation. In order to maintain energy homeostasis, highly glycolytic cancer cells lead to glucose deprivation in the extracellular space by consuming large amounts of glucose (and glutamine). Low-glucose conditions in the tumour microenvironment, in turn, can cause a loss of stromal caveolin-1, yielding oxidative stress which mimics hypoxia (‘pseudohypoxia’) through activation of HIF-1 and NF-kB [29]. NF-kB has been shown to be a direct modulator of HIF-1 expression in inflammation and hypoxia [30,31], and in osteosarcoma, NF-kB upregulation has been demonstrated to be induced by acidosis [32], supporting the idea that acidosis and hypoxia can reciprocally modulate each other’s behaviour.

### 2.2. Proton Extruders in Bone Sarcomas

Cytosolic acidification is extremely toxic to both normal and cancer cells, eventually leading to apoptosis [33]. Sarcoma cells get rid of excessive intracellular proton accumulation through extruders and transporters located on the plasma membrane or lysosomal membrane, which strongly acidify the extracellular space via direct pumping/transport or by exocytosis, respectively [34].

Previous reports on extracellular acidification in bone sarcomas have made use of preclinical in vitro models and various techniques to measure pHe, such as the use of macro- or microelectrodes or the measurement of extracellular acidification rate (EACR) values by Seahorse technology. These techniques have shown that the activity of these extruders/transporters is responsible for strong acidification of the medium, both in the extracellular space and near the cell membrane [16]. Additionally, the enhanced acidification ability in stem cells derived from a soft tissue sarcoma has been demonstrated using acridine orange and lysosensor staining [16,34,35].

Among the most studied proton exchangers and transporters, sarcomas express certain subunits of vacuolar H^+^-ATPase (V-ATPase), such as V_1_B_2_ and V_0_c V-ATPase subunits, the Na^+^/H^+^ exchanger isoform 1 (NHEs, mainly NHE1), the monocarboxylate transporters (MCTs, mainly MCT1, also known as lactate–proton symporter), the Na^+^-dependent Cl^−^/HCO3^−^ exchanger, and carbonic anhydrases (CAs) isozymes, mainly CAII, CAIX and CAXII [36]. Studies describing the expression and the role of these molecules in the extracellular acidification and behaviour of bone sarcomas are reported in Table 1, Table 2, Table 3 and Table 4.

Several drugs have been tested to target these ion extruders/transporters as anticancer therapy. For a more extensive discussion, see Section 5.1.

The most studied ion/proton extruders/transporter is the V-ATPase, followed by the CAIX enzyme. V-ATPases are ubiquitous proton pumps that are found either on the intracellular membranes, such as lysosomes, or, for specialised cells, at the plasma membrane. V-ATPases use the energy of ATP to transport protons from the cytosol to intracellular compartments or to the extracellular space. The V-ATPase consists of an ATP-hydrolytic domain (V_1_) and a proton-translocation domain (V_0_) [49]. Its energy-consuming activity requires the close association of all the components of the complex, which is provided by the C-loop [50]. Studies on V-ATPase expression and activity in bone sarcomas are mainly related to the analysis of preclinical models and, less frequently, of tissue samples.

CAIX is one of the 15 carbonic anhydrase isoforms present in humans, among which 12 are functional [51]. Carbonic anhydrases are a large family of dimeric zinc metalloenzymes with an extracellular active site that catalyses the reversible hydration of carbon dioxide to carbonic acid and are involved in respiration and acid–base balance, facilitating acid secretion in different cell types [52]. Evidence for CAIX expression in bone sarcomas has been largely based on the analysis of human tissue samples.

A less considered but important acid extruder is the voltage-gated proton channel (Hv1). This has been found to be expressed in the cells of origin of bone sarcoma, the mesenchymal stromal cells (MSC). Its pharmacological inhibition in MSC significantly decreases cell differentiation and mineral matrix deposition [53]. However, although Hv1 expression has been demonstrated in different cancers that frequently colonise bone, including breast and colorectal carcinomas [54,55], no data have been reported in bone sarcomas. In this context, it might be interesting to compare the ability of bone sarcomas to acidify the extracellular space with respect to other types of cancers that are able to expand in bone, such as bone metastases (BM) (see ref. [50]); carcinoma cells metastasizing to the bone share with bone sarcoma cells different mechanisms of proton extrusion, including the expression of V_1_B_2_ and V_0_c V-ATPase subunits, CAIX, MCT1, and MCT4. As an example, we have recently found mRNA expression of CAIX in breast and renal carcinoma cell lines, with a significant increase under reduced oxygen conditions with respect to normoxia [50,56]. Additionally, different isoforms of V-ATPase, including the V_1_C_1_ [57] and the V_1_B_2_ and V_1_G_1_ subunits [58], are expressed by breast carcinoma cells with a specific tropism for bone. Finally, it has been demonstrated that the expression of MCT4 in tumour cells is responsible for a metabolic coupling with bone-resorbing osteoclasts, thereby inducing a higher osteolytic activity in BM from breast carcinoma [18].

In summary, several lines of evidence suggest that an increased glycolytic rate and subsequent activation of several ion extruders and transporters in different cancer cells that grow in bone are the main causes of tumour interstitial acidosis.

### 2.3. Bone Resorption as a Source of Extracellular Acidification

In the bone soil, to expand and invade the surrounding normal tissue, sarcoma cells degrade the hard extracellular matrix by directly or indirectly stimulating the activity of osteoclasts, the highly specialised bone-resorbing cells. The bone microenvironment is a fertile ground for tumour growth. Under physiological conditions, the process of bone remodelling couples osteoclast-mediated bone resorption and osteoblast-promoted bone formation to maintain bone homeostasis. However, the development and progression of primary bone tumours, including osteosarcoma, severely disrupt this balance and induces a ‘vicious cycle’ between osteoclasts, osteoblasts, stromal cells, and cancer cells. In the bone soil, in order to expand and invade the surrounding normal tissue, sarcoma cells degrade the hard extracellular matrix by directly or indirectly stimulating the activity of osteoclasts that resorb bone, as well as directly eroding bone through the secretion of metalloproteinases (MMPs). The induction of osteoclast activity can be triggered by a plethora of growth factors that also commonly regulate physiological bone remodelling and can be secreted by cancer cells, or by tumour-stimulated osteoblasts. Of these, the most important is the receptor activator of nuclear factor-kappa B ligand (RANKL). Other factors, such as interleukin 1 (IL-1), interleukin 6 (IL-6), tumour necrosis factor-alpha (TNFa), parathyroid hormone-related protein (PTHrP), or transforming growth factor-beta (TGFb), mediate RANKL receptor (RANK) expression on the surface of osteoclasts, thereby favouring osteoclast maturation and activation [59]. Furthermore, we have recently demonstrated that a low pH further induces osteoclast activity, both directly and indirectly, by stimulating osteoblasts to secrete pro-osteoclastogenic paracrine mediators such as IL-8 and IL-6 [56].

Once stimulated, mature osteoclasts can resorb bone through a multistep dynamic process. First, osteoclasts migrate and attach to the bone surface that is to be degraded and removed, thus forming a tight ‘sealing zone’. Then, the plasma membrane polarises to form the resorption organelle, the ruffled border, a unique folded highly permeable membrane facing to the bone surface to be resorbed [60]. Subsequently, to dissolve the mineralised component of bone, osteoclasts secrete hydrochloric acid into the resorption lacunae (Howship’s lacunae) mainly via plasma membrane V-ATPase (a3 isoform) [61]. Proton pumping performed by osteoclasts during bone resorption activity is an energy-consuming intensive process that relies primarily on the glycolytic metabolism of osteoclasts [62]. It is noteworthy that the expression of a3 is 100-fold higher in osteoclasts than in other cell types [63]. The activity of V-ATPase is also coupled with the activity of the chloride ion–proton channel antiporter ClC-7 [64], and both proteins are clustered in the ruffled border domain.

Finally, as an additional player in the acidification activity of osteoclasts, it has been demonstrated the expression of Hv1 that helps proton release and bone mineral dissolution, thereby promoting bone resorption [65,66,67].

As a consequence of the proton extrusion activity, in Howship’s lacunae, the pH reaches very low values, around 4.5 [60]. At the end of the resorption process, protons pumped into Howship’s lacunae diffuse in the extracellular space, thus causing further acidification of the tumour microenvironment. Adversely, proteinaceous component of the matrix, mainly type I collagen, is degraded through the activity of the osteoclast-derived cysteine proteinase cathepsin K, which is responsible for the breakdown of collagen I, osteopontin, and osteonectin [68].

Osteoclast differentiation and activity result in dysregulated bone lysis and release of bone matrix growth factors such as TGFb, insulin-like growth factor 1 (IGF1), fibroblast growth factor (FGF), or bone morphogenetic protein (BMP), which, in turn, can promote tumour cell proliferation and further bone destruction [69,70]. This ‘vicious cycle’ between cancer cells and the bone microenvironment was first described in bone metastasis, but in fact, there is evidence supporting the notion that osteosarcoma cells, for example, mediate bone destruction by stimulating osteoclast differentiation and activity as bone metastasis [71,72]. In addition to osteoclasts, acid-mediated resorption of the bone mineralised matrix can also be performed by osteocytes. Osteocytes are the final fully differentiated form of osteoblasts that are trapped in the hard matrix and directly remodel the bone walls of their lacunar–canalicular systems in a process known as perilacunar/canalicular osteocytic remodelling. As with osteoclasts, this process relies on the combined activity of MMPs, vacuolar acid-secreting H^+^-ATPases [73,74], and other enzymes, such as cathepsin K and carbonic anhydrases [75]. However, the interaction between sarcoma cells and osteocytes is completely unexplored, and it is still unknown whether perilacunar remodelling can be induced by invading cancer cells.

Finally, in the context of the acid extracellular tumour microenvironment, it should be noted that an excess of extracellular protons may also modulate the activity of cation channels, including calcium receptors [76,77]. Calcium (Ca^2+^) signalling is crucial, both for bone physiology and sarcoma progression. Indeed, both osteoblasts and osteoclasts, as well as osteosarcoma cells, express calcium-sensing receptors on the cell membrane [78,79], and Ca^2+^ is an essential mediator for cell differentiation, bone resorption, and gene transcription in osteoclasts, and for the aggressiveness of tumour cells [80,81]. However, the interference of extracellular acidosis in Ca^2+^ signalling in bone sarcoma is an unexplored field of research. For a more detailed discussion of the potential effect of high H^+^ extracellular concentration on cation channels that are expressed by bone sarcomas, see Section 4.1.

In conclusion, these findings demonstrate that, in addition to tumour cells, tumour-induced bone-resorbing cells of the bone microenvironment contribute to acidify the microenvironment of sarcomas.

## 3. Effect of Acidosis on Sarcoma Cells

The extracellular acidification derived from cancer cells and from the tumour-associated stroma is responsible for the modulation of bone colonisation by sarcoma cells. Indeed, an acidic pHe promotes cancer invasion, survival, and angiogenesis, and alters the cell permeability to anticancer drugs by many different mechanisms, thereby preventing their effective targeting.

Extracellular acidosis has also been described to influence anticancer immune response and autophagy in a number of solid tumours. However, the role of autophagy in mediating survival to acidosis has not been confirmed in osteosarcoma, where the autophagic flux seems to be unchanged between pH conditions (7.4 and 6.8) [37,82], and the impact of acidosis on the infiltration of inflammatory or immune cells has not been explored thus far. Furthermore, the system by which tumour cells can sense extracellular acidosis has not been deeply investigated yet, but few specific sensors have been identified. Finally, it is important to bear in mind that preclinical studies that investigated on sarcoma acidic microenvironment and based on cell culture medium acidification have high heterogeneity of pH values: in most cases, the studies were carried out with a pH range between 6.5 and 6.8 [22,24,25,26], but in other cases, harsher experimental conditions were used (pH 5.8 in [9]). On the other hand, the development of a 3D model has led to the development of physiological pH culture values by using an unbuffered culture medium, thus allowing 3D spheroids to adapt the pH value to their own metabolism [54]. Thus, these different experimental systems and different pH values might have led to different outcomes.

### 3.1. pH Sensors in Sarcoma Cells

In the TME, different ion channels behave not only similar to ion transporters but also similar to sensors and transducers of altered pH as they can be affected by both extracellular and intracellular pH [83]. Furthermore, they greatly contribute to cancer progression [84,85,86,87]. As an example, in osteosarcoma, the voltage-gated potassium channel Kv1.3, transient receptor potential cation channel subfamily M member 8 (TRPM8), and piezo type mechanosensitive ion channel component 1 (Piezo1) are among the most expressed pH-sensitive ion channels and correlate with tumour progression [88,89,90].

On the other hand, the high concentration of protons in TME may also strongly affect the biological functions of these pH-sensitive proteins and receptors, since it may induce Kv1.3 potassium channel inactivation, and the alteration of the signalling pathway mediated by the Ca^2+^-permeable channels, TRPM8 and Piezo1 [91,92,93], ultimately altering their proaptoptotic signalling. However, the acid-mediated effect on these ion channels and the downstream signalling has never been explored in sarcoma.

### 3.2. Effect of Extracellular Acidosis on Tumour Invasion, Survival, and Metabolism

Invasion occurs through invadopodia, dynamic actin-rich membrane protrusions that penetrate within the extracellular matrix and degrade it through the spatial and temporal release of proteases and protons [94]. The protonation of the matrix metalloproteinases is dependent on the activation of the proteinases and requires the redistribution and activation of V-ATPases and NHE1 to the tip of the invadopodia. Thus, local invasion is strongly modulated by the acidification activity of these proton/ion transporters and by the presence of an acidic pHe.

In sarcomas, the acidic microenvironment activates survival pathways and increases migration and invasive potential [16,37]. However, further molecular mechanisms are responsible for the acidosis-mediated progression of sarcomas. In osteosarcoma, we demonstrated the pH-dependent activation (at a pH of 6.5) of a stress-regulated switch that promotes the recruitment of the TNF-receptor-associated factors/cellular inhibitor of apoptosis protein 1 (TRAF/cIAP) complexes, and nuclear factor kappa-light-chain-enhancer of activated B cells (NF-κB) pathway [95]. This activation ultimately leads to an increase in cancer cell survival, suggesting a role for TRAF/cIAP proteins as promising targets for anticancer therapy. As an in vivo confirmation of the intimate association between acidosis and cancer cell survival, we found a significant correlation between V-ATPase and TRAF1 or NF-κB1 expression in tissues from osteosarcoma xenografts.

More recently, evidence has shown that extracellular acidosis, obtained in unbuffered conditions, is also responsible for prominent metabolic plasticity that leads to the accumulation of intracellular lipids, specifically sphingolipids and sphingosine 1-phosphate (S1P). Impairing S1P levels by means of Fingolimod, an FDA-approved drug for the treatment of multiple sclerosis, was of predominant importance to decrease the migration potential of acid-resistant cells, to increase apoptosis, and to impair xenograft growth [82]. This suggests, for the first time, the use of an anticancer drug that has the potential to specifically target the acid-resistant subpopulation in osteosarcoma.

Finally, by studying both standard monolayer cultures and cancer stem cells and by extensive metabolomic analysis, we demonstrated that extracellular acidosis completely remodels cancer cell metabolism by inducing glycolysis repression and by increasing the amino acid catabolism and the urea cycle [96].

### 3.3. Effect of Extracellular Acidosis on Tumour Sensitivity to Anticancer Drugs

Tumour acidosis is also a major cause of drug resistance and therapeutic failure. First of all, a low pHe (pH 6.5) significantly decreases the growth rate of cancer cells, thereby affecting the IC50 values of drugs that target actively proliferating cells [37]. However, an acidic pHe may also impact the response to therapeutics through additional complex mechanisms. The pH gradient across cellular membranes is crucial for determining the passive diffusion of small molecules. ‘Ion trapping’ (or pH partitioning) is the physiological process regulating passive permeability through the cellular membrane of negatively or positively charged compounds, such as ionisable compounds containing weak bases or weak acids. The lysosomal and the cytoplasmic membranes can compartmentalise drugs and, as a consequence of the pH partitioning, drugs can be hindered from reaching their molecular target because they become trapped on the wrong side of cellular membranes. The extent of ionisation for a molecule depends both on its intrinsic pKa values(s) and the pH of the solution. In an acidic extracellular microenvironment, weak bases will be positively charged to a larger extent, thus influencing the diffusion of the drug inside the target cells [14].

The cellular membranes that can compartmentalise drugs are both the cytoplasmic membrane and the lysosomal membrane. Acidic lysosomes can sequester weakly basic molecules from the cytosol to an extent that is directly related to the level of lysosomal acidosis, thereby preventing the drug targeting [97]. In this context, it is noteworthy that a high extracellular concentration of protons increased both the number and the acidification of lysosomes in osteosarcoma cells [37].

Additionally, the cytoplasmic membrane contributes to the ‘ion-trapping effect’. In the presence of an acidic extracellular microenvironment, weakly basic drugs are forced to stay outside the cell. We confirmed this mechanism for doxorubicin in osteosarcoma cells [37]. Conversely, the presence of a low extracellular pHe allows for the permeability of weakly acidic drugs. In such a case, the neutral form of a weakly acidic compound may be favoured, and the uncharged species can freely diffuse across the plasma membrane. Since the cytosolic pH is slightly alkaline, once the acidic drug has crossed the plasma membrane and entered the cell, it is ionised and trapped within the cell. In this case, the cytotoxic activity may be enhanced by extracellular acidosis. Known examples of anticancer drugs containing weak acids are 5-fluorouracil and cyclophosphamide [14]. However, preclinical studies on the comparison of cytotoxicity at different pH values of these drugs in inhibiting bone sarcomas have never been performed.

### 3.4. Effect of Extracellular Acidosis on Tumour Angiogenesis and Others

The anarchic formation of new vessels that provide O_2_ and nutrients needed by actively proliferating cells is induced by tumour cells through the release of pro-angiogenic factors, such as vascular endothelial growth factor (VEGF) and interleukin 8 (IL-8) [98], or through the stabilisation of HIF-1 that are promoted by extracellular acidosis [99,100]. Interestingly, in osteosarcoma cells under acidic conditions, we observed increased release of extracellular nanovesicles with proangiogenic activity, including urokinase-type plasminogen activator (uPA), angiopoietin-2 (Ang-2), and VEGF, as well as the presence of miRNAs related to angiogenesis, as demonstrated by the formation of tubule branches in the chorioallantoic membrane (CAM) [101], suggesting that local acidosis might be responsible for promoting neoangiogenesis.

## 4. Effect of Acidosis on Different Cells and Elements of Sarcoma Microenvironment

Cancer cells are not solely responsible for the growth of cancer and the spread to distant organs. A complex structure, formed of cancer cells that directly interact with stromal cells under different microenvironmental conditions, constitutes the bulk of the tumour. Among stromal cells, the microenvironment of bone sarcomas includes MSC, osteoblasts and osteoclasts, cancer-associated fibroblasts, and immune cells: all these different cell types coexist and infiltrate the tumour [102,103]. In particular, similar to physiological wound healing, MSCs are recruited from the bloodstream to the site of the tumour lesion, where they contribute to the rapid tumour expansion [68]. MSCs are crucial for the initiation [104], as well as the progression of the lesion [105]. However, in the context of mesenchymal tumours, MSCs are hardly distinguishable from tumour cells.

**Figure 1:**
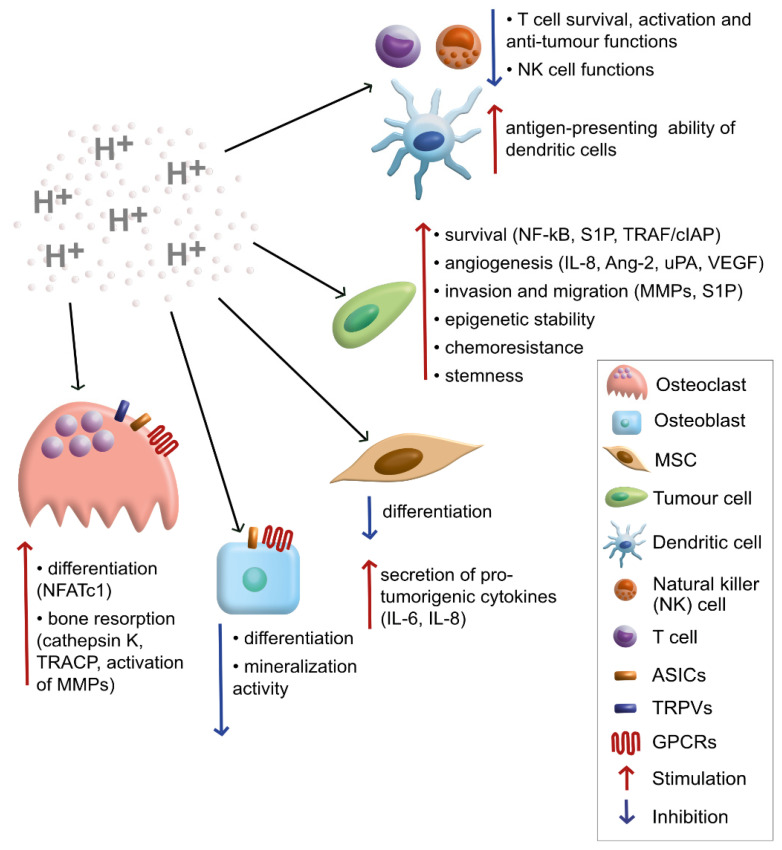
Graphical summary of the effects of extracellular acidosis on cells of the bone sarcoma microenvironment.

Importantly, cancer cells are not the only population being affected by extracellular acidosis. The effects of a low pHe are observed also on stromal cells of the bone microenvironment, and these may, in turn, indirectly modulate the behaviour and the aggressiveness of tumour cells (Figure 1).

### 4.1. Bone Cells Sense and React to Extracellular Acidification

It is widely recognised that local variations of pHe greatly impact osteoblast and osteoclast differentiation and activity. Thus, as in other pathological conditions (i.e., inflammation), in the altered tumour microenvironment, bone cells can perceive acidosis and react to such stress signals by modulating their activities, as well as through paracrine communication by stimulating cancer progression.

Cells of the osteogenic lineage react to a high extracellular concentration of protons by impairing their osteogenic activities, namely, osteoblast differentiation, matrix deposition, and mineralisation [106,107]. Adversely, in osteoclasts, a low pHe increases the formation of resorption pits (maximal stimulus at pH < 6.9 [108]) and upregulates the activity of cathepsin K, tartrate-resistant acid phosphatase (TRACP), and TNF-receptor-associated factor 6 (TRAF6) [109,110,111].

Bone cells sense pH changes through specific proton sensors and channels that are typically expressed by sensory neurons. Among them, the acid-sensing ion channels ASIC2, also known as amiloride-sensitive cation channel 1, neuronal (ACCN1), and ASIC3/ACCN3 are mostly abundant in bone. Specifically, previous reports have shown the expression of ASIC1/ACCN2, ASIC2/ACCN1, and ASIC3/ACCN3 mRNAs [112] in human osteoblasts. Besides ASICs, metabotropic proton-sensing G protein-coupled receptors (GPCRs) have also been recently identified as proton-sensing machinery in osteoblasts [113,114,115]. Similarly, we recently found that MSC, osteoblasts, and CAF express ASIC4/ACCN4, ASIC3/ACCN3, G protein-coupled receptor (GPR)-65, and GPR4 at levels comparable to or even higher than those expressed by cells of neuronal origin and that in MSC, the incubation with an acidic medium increases the expression of ASIC4/ACCN4 and GPR65 [58].

Regarding the osteoclastic lineage, human monocytic osteoclast precursors express ASIC1/ACCN2, ASIC2/ACCN1, and ASIC3/ACCN3. This expression persists also after the induction of osteoclast differentiation, albeit at a lower level. Likewise, transient receptor potential vanilloid (TRPV) channels, which are typically expressed by sensory neurons, and the ovarian cancer G protein-coupled receptor 1 (OGR1) which belongs to the GPCR family are proton sensors and have been involved in osteoclast differentiation and survival [50]. In particular, TRPV1, TRPV2, and TRPV4 channels are crucial for osteoclast biology [80,116,117]. Notably, TRPVs are activated also by severe acidosis (pH 5.4) [118] and TRPV4 seems to be the major mediator of the acidosis-induced osteoclast formation as its antagonist, RN1734, partially inhibited the pH-dependent osteoclastogenesis, while its agonist 4-α PDD enhanced osteoclast formation under mild acidosis [117].

Under this context, it is noteworthy that both ASICs and TRPVs are also permeable to cations other than H^+^, like Ca^2+^ and the reciprocal interactions between H^+^ and Ca^2+^, and the competition of H^+^ for the same binding-site of Ca^2+^ may modulate the activity of these pH sensors and, thus, the downstream biological effects. Specifically, for ASICs, due to the binding competition, Ca^2+^ binding favours the closed state, and H^+^ binding leads to the open state [119]. Furthermore, increased extracellular Ca^2+^ concentration can significantly decrease the pH sensitivity of ASIC1 and ASIC3 [120]. Thus, this strong interplay between H^+^ and Ca^2+^ may occurs also in normal bone cells in the sarcoma microenvironment: it is already well known that TRPVs mediate Ca^2+^ signalling and are produced in mature osteoclast differentiation to sustain the intracellular Ca^2+^ level for the maintenance of active NFATc1 that regulates terminal cell differentiation [121], and the presence of an excess of protons in the sarcoma TME may interfere with Ca^2+^ signalling mediated by TRPVs in osteoclasts and may directly alter the osteoclast physiology and activity.

Overall, these data indicate that normal bone cells perceive and react to the acidification of the bone sarcoma microenvironment. The ultimate result is an unbalance of bone remodelling. In conclusion, a low pHe appears to be an essential requirement for the initiation of the osteolytic process, but it may also be involved in altered bone formation as it occurs in osteogenic sarcomas such as osteosarcoma.

### 4.2. The Acid-Stimulated Secretome

Tumour-derived acidosis may favour tumour expansion by reprogramming stromal cells to the secretion of proinflammatory cytokines. Decrease of local pH is per se an inflammatory stimulus that causes the release of various enzymes during phagocytosis, the damage of vasculature and other surrounding tissues, and the prolonging of the healing process by stimulating new inflammatory reactions [29]. We have recently shown that extracellular acidosis directly activates the NF-kB inflammatory family of transcription factors and thus the secretion of NF-kB-related cytokines, chemokines, and growth factors by the osteosarcoma-associated stromal compartment formed by osteoblasts, MSC, and CAF [32,58]. Regardless of the source of acidosis, after a few hours, incubation with a pHe 6.8 activates RelA, RelB, or NF-kB that, in turn, induce the expression of the inflammatory cytokines IL-8 and IL-6 and enhances cancer stemness (formation of spheroids and expression of the stemness-related markers oct4 or Nanog [32]). In the same study, by using a blocking antibody against the IL-6 receptor, we demonstrated that acid-induced release of IL-6 by normal mesenchymal cells was directly responsible for bone cancer migration and invasion [32]. Intriguingly, IL-6 secretion seems to be directly dependent on the acid-stimulated MSC, whereas the tumour cells contribute little to the release of paracrine tumour-stimulating factors under acidic pH conditions. This is of note because it highlights the importance of the stromal subpopulation in enhancing cancer progression. Furthermore, the exposure of osteosarcoma cells to the secretome of acid-stimulated MSC reduced the toxicity of doxorubicin and thus promoted the development of a chemoresistant phenotype [32].

Altogether, these observations warrant the role of local acidosis in promoting a protumourigenic phenotype in bone sarcomas also by inducing a proinflammatory and a pro-osteolytic secretome by cells of the osteogenic lineage.

### 4.3. Matrix Remodelling/Degradation

Another important feature of the sarcoma microenvironment is the composition and organisation of the ECM, whose mechanical properties affect cancer cell behaviour and that may be, in turn, influenced by tumour-derived extracellular acidosis. ECM is mainly secreted by stromal cells, and it is composed of various macromolecules, including collagens, glycoproteins (fibronectin and laminins), proteoglycans, and polysaccharides [122]. ECM is also secreted from tumour cells, especially from osteogenic sarcoma. However, how low extracellular pH affects the synthesis and secretion of proteins of ECM is an almost uncovered field of investigation. During cancer progression, an excessive ECM remodelling occurs by proteinase activity, such as MMP-2 and MMP-9, and small ECM fragments are released into the circulation [123]. In melanoma cells, it has been reported that acidic culture conditions induce the increase of 103-kDa gelatinase/type IV collagenase secretion [124]. Furthermore, the membrane-bound MMP-14 has an acidic pH optimum and has been observed to be in close association with CAIX in invadopodia [125]. Additionally, in sarcoma, with particular regard to Ewing sarcoma, we previously found an increase of MMPs activity when tumour cells were cultured at low pH, as evaluated by gelatine-quenching assay and an increase of the ability to degrade type I collagen [16]. However, with the exception of the mentioned report, no other data have been published about the correlation between acidosis and ECM remodelling/degradation in bone sarcomas.

### 4.4. Effect of Extracellular Acidosis on Immune Reactivity to Cancer Cells

In the sarcoma microenvironment, different cells and cytokines of the immune system may be included, such as tumour infiltrating lymphocytes (TILs) and associated macrophages, expression of immune checkpoint inhibitors such as cytotoxic T-lymphocyte-associated protein 4 (CTLA-4), programmed cell death-1 (PD-1), and programmed death-ligand 1 (PD-L1), and major histocompatibility complex (MHC) antigen expression [126]. All of these components may be important for prognosis and responses of tumours to immunologically targeted therapies and are potential therapeutics or therapeutic targets. However, although significant progress in the field of immunotherapy, particularly as regards the clinical use of immune checkpoint inhibitors, has been made [127], durable response rates remain low [128], and current sarcoma immunotherapies still fails to induce an antitumoural response [129] implying that other immunosuppressive activities or effects are possibly present. Among these, tumour-derived extracellular acidosis may have an unexplored role. Indeed, in other types of cancer, the formation of an acidic microenvironment represents an efficient tumour strategy and forms such as an immune sanctuary to overcome immune surveillance since it profoundly alters the functions of cells of the immune system, including T cells, neutrophils, macrophages and dendritic cells (DCs) [130,131]. In particular, both cancer and immune cells are highly dependent on the glycolytic pathway for survival, proliferation, and activity. An increased rate of glycolysis, as it occurs in cancer environment, leads to a significant decrease in glucose availability and, although cancer cells can enter quiescence in the absence of glucose, activated T cells are not able to survive without glucose when attempting to expand into an acidic environment [132]. Notably, a high extracellular concentration of protons impairs glycolysis per se [96,133]. Furthermore, an acidic pH blocks the activation and antitumour functions of T cells in vitro through sequestration of interferon-gamma (IFN-γ) [134]. Tumour acidity also promotes tumour progression by negatively affecting the maturation and function of Th1 lymphocytes while stimulating the progression of tumour-promoting Th2 lymphocytes by inactivation of IFN-γ and suppression of tumour necrosis factor-α [132]. Finally, several lines of evidence have suggested that the contribution of extracellular acidosis to cancer growth is related to both the suppression of T cell function and to modulatory effects on additional cells of the immune system. In particular, Husain et al. demonstrated that tumour-derived lactate inhibits natural killer (NK) cell function, both directly and indirectly, i.e., by increasing the numbers of myeloid-derived suppressor cells (MDSCs) that, in turn, inhibit NK cytotoxicity [135]. A low pH also reprogrammes tumour-associated macrophages (TAMs) into a proangiogenic phenotype [136], activates neutrophils [137,138], and improves the antigen-presenting capacity of DCs derived from murine bone marrow [139]. However, no investigations have been performed thus far on sarcomas in this regard. Future studies will help find possible novel approaches to improve the outcomes of immunotherapy in sarcoma patients.

## 5. Targeting Acidosis in Bone Sarcomas

The use of preclinical models that can mimic the extracellular acidic sarcoma microenvironment and the selection of assays that are not technically affected by the presence of an acidic pH have been fundamental for the identification of novel targets and the development of effective therapeutic strategies against acidosis in cancer. In vitro, monolayer models have to face the caveat of acidic pH being adjusted by a buffer solution that cannot be regulated throughout the experiment. In 3D experiments, spheroids or organoids grown in unbuffered media can, instead, adjust the pH to their own metabolic features and to the intrinsic acidification processes. The latter method has the advantage of resembling the physiological pH regulations seen in vivo. Additionally, the expression and the activity of reporter or housekeeping genes that are commonly used to study the induction or the inhibition of specific targets or proteins, such as b-actin or the green fluorescent protein (GFP), can be strongly affected by an acidic microenvironment. A recent paper has highlighted that among the most commonly used housekeeping genes, only YWHAZ, GAPDH, GUSB, and 18S rRNA are stable throughout pH modifications [140]. Furthermore, scientists working in this field of research should be aware that the fluorescence of wild-type GFP is stable from pH 6 to 10 but decreases at pH < 6 and increases from pH 10 to 12 [141]. The pH stability of GFP can also be exploited for specific purposes: for example, the superecliptic pHluorin (SEP) is a mutant GFP widely used in vitro as a pH reporter, as it is nearly nonfluorescent at pH 6 but brightly green at pH 7.4 [142].

Additionally, in vivo, the assessment of pH imaging methods is invasive, costly, or requires long acquisition times, and in some cases may not be suitable for high-throughput preclinical animal studies. Imaging methods include CEST-MRI, a quantitative method that accurately recapitulates tumour pH maps [143], or pH-sensitive ratiometric reporters such as pHLuc [142]. Despite the limitations, these imaging methods are of crucial importance in the assessment of therapeutics based on targeting cancer acidosis.

The therapeutic strategies that have been developed to target cancer acidosis are based on several approaches, namely, (1) hampering of proton extruders/ion transporters; (2) targeting cancer cell lysosomes through the use of photodynamic therapy; (3) use of inhibitors of acid-sensing ion channels that can possibly hinder the activation of the tumour-associated stroma (see Section 4.1). However, the last class of drugs has been extensively studied only as analgesic and anxiolytic drugs, and as drugs for the treatment of ischemic stroke [144], but has never been considered thus far for the treatment of sarcomas. Finally, recent evidence has highlighted molecular pathways that are selectively activated in acidic-treated cells. These pathways can regulate oncogenes or oncometabolites or be involved in the generation of bioactive lipids. In the former case, the RAB39A-RXRB axis has been shown to have a prominent role in the development of osteosarcoma stemness and aggressiveness at a pH of 6.5 [145], while in the latter case, the pH-dependent accumulation of S1P seems to be of paramount importance in the survival and growth potential of osteosarcoma xenografts [82].

### 5.1. Hampering Proton Extruders/Ion Transporters

Several drugs have been developed to target ion extruders/transporters as anticancer therapy. Inhibitors of the V-ATPase and CAIX have been the most explored for treating sarcomas. Studies considering these two approaches are listed in Table 1 and Table 2.

To specifically target the V-ATPase, siRNA or Bafilomycin have been taken into account; nonetheless, their use can be hardly translated to the clinic for their instability or high toxicity, respectively. On the contrary, the use of proton pump inhibitors (PPIs), such as omeprazole or esomeprazole, has been extensively investigated. PPIs are acid-activated pro-drugs that reduce gastric acid production by inhibiting the H^+^/K^+^-ATPase pump and have been successfully used for the treatment of peptic disease [146]. Intriguingly, when used at high concentrations, PPIs can also effectively inhibit the activity of V-ATPase [147,148]. In preclinical models of bone sarcomas, although tumour growth was unaffected, treatment with a high concentration of PPI significantly increases the sensitivity to doxorubicin [16,37,95] Finally, in a multicentre trial on human patients, pretreatment with omeprazole increased the local cytotoxicity of standard chemotherapy, as expressed by the increased percentage of tumour necrosis. This was particularly evident in chondroblastic osteosarcoma, an histological subtype that normally shows poor histological response [149].

CAIX targeting has shown successful results with the use of sulphonamide-derived inhibitors. Among them, a compound obtained starting from benzenesulphonamide derivatives (covered by patent) has been successfully used to inhibit tumour growth in a xenograft model of osteosarcoma. Although not well investigated yet, the use of this compound is quite promising, since, among the different CA isoforms, CAIX appears to be highly and selectively expressed in cancer cells, concomitantly implying less toxicity and an increased selective anticancer effect [40]. In a recent paper, Tauro et al. have developed a dual CA/matrix metalloproteinase inhibitor incorporating a bisphosphonic acid, which increases selective anticancer targeting [150]; this drug possibly and directly targets tumour-induced osteolysis by combining a cargo molecule of bisphosphonate that delivers a blocker of MMP-mediated invasion and an inhibitor of CAIX-mediated acidification to the site of osteolysis.

Regarding the use of MCT1 and NHEs inhibitors for the treatment of bone cancers, very few in vitro data have been reported (see Table 3 and Table 4), with the exception of the use of [alpha]-Cyano-4-hydroxycinnamate (CHC) that, in an orthotopic model of osteosarcoma, strongly impaired both chemoresistance and tumour growth [46].

### 5.2. Targeting of Cancer Cell Lysosomes by Photodynamic Therapy

Photodynamic therapy (PDT) is defined as the photo-induced irreversible destruction of abnormal cells and is based on the uptake of a photosensitiser molecule which, upon being excited by visible or near-infrared light, reacts with oxygen and generates reactive oxygen species (ROS) in target tissues, leading to cell death. PDT is therefore a minimally invasive anticancer modality with low-power light energy. ROS comprise singlet oxygen, superoxide anion, and radicals that generate from the conversion of molecular oxygen that reacts with the triplet state of the photosensitiser that is formed via photoexcitation. The generated free ROS oxidise biological substances, including nucleic acids, lipids, and proteins, leading to severe alterations in cell signalling cascades or in gene expression regulation and to activation of death-promoting physiological responses.

As discussed in Section 2.2, to avoid intracellular acidification, the excess of protons in the cytosol of tumour cells may be pumped into the lumen of the lysosomes, thereby decreasing the intra-organelle pH [16,151,152]. Acridine orange is a fluorescent cationic dye originally known as a detector of bacteria and parasites and an antimalarial drug. More recently, it has been described as an anticancer agent [153]. Since it has a low molecular weight, acridine orange easily diffuses into interstitial tissues and the cytoplasm and, due to protonation, accumulates into intracellular acid vesicles, leading to the formation of membrane-impermeable monomeric, dimeric, or oligomeric aggregates [16,151,152]. Acridine orange has thus a strong and selective tropism for tumour cells, as tumour cells have more acidic vesicles than normal cells because of their specific ability to effectively reduce the excess of protons in the cytoplasm by active transport across the plasma membrane and storage within the lysosomal compartment [154]. Furthermore, when photo-activated by blue light (466.5 nm) [155], or exposed to low-dose (1–5 Gy) X-ray irradiation [156], it generates singlet oxygen (1O2) thereby acting as an acid-targeting photosensitiser. The formed reactive species oxidise the fatty acids of the lysosomal membrane, causing the leakage of lysosomal enzymes and protons, followed by cell death [157].

To date, several data have demonstrated that acridine orange exerts selective cytocidal effects on tumour cells, showing no toxicity on normal cells. Furthermore, in the last 20 years, a combined technique of PDT and radioactivation (RDT) of acridine orange has been successfully developed and applied to clinical cases, demonstrating excellent outcomes in terms of inhibition of local recurrence and preservation of limb function after intra- or marginal tumour resection. These studies include humans affected by bone sarcomas, although the same type of approach has been tested in companion animals with spontaneous fibrosarcoma [158,159,160,161,162]. Specifically, following marginal or even intralesional gross removal of the tumour, it was possible to selectively target residual sarcoma and spare the surrounding normal tissues, with a satisfactory functional result. The procedure is safe without local or systemic complications. This technique proved to be particularly advantageous in sarcomas arising around the forearm and a valid alternative to wide surgical resection followed by limb reconstruction, without increasing the local recurrence rate [163]. Systemic administration of acridine orange with low-dose radiation therapy is also under evaluation for nonresectable bone sarcomas. The procedure appears to be safe and preliminary results are encouraging.

Talaporfin, also known as aspartyl chlorin, mono-L-aspartyl chlorin e6, NPe6, or LS11, is another photosensitiser that can target lysosomes and has been proposed for the treatment of bone sarcoma in addition to acridine orange [164,165,166]. Talaporfirin is uptaken by sarcoma cells through a KRAS-dependent endocytotic process. However, the correlation between its selective targeting and the degree of lysosomal acidification has not been unveiled yet.

## 6. Conclusions

After over 10 years of research, the crucial role of acidosis in bone sarcoma growth and progression has been clearly established. However, the development of acid-targeted drugs for the treatment of bone sarcomas is still in its infancy. To date, most of the drugs targeting ion/proton extruders and transporters have failed to be translated to clinical trials. One possible explanation is the redundancy of cellular systems controlling pHe. Thus, their targeting is quite challenging: it can easily turn to be ineffective, or when it works, extremely toxic. Nevertheless, given the relevance of intratumoural acidosis in bone cancers, the use of CAIX inhibitors, acid-targeted PDT strategies, or novel drugs that can safely and selectively impair the protumourigenic pathways that are selectively induced by extracellular acidosis may hold, for the future, helpful results to improve patient survival.

## Figures and Tables

**Table 1:** V-ATPase expression and targeting in bone sarcomas.

Type of Cancer	Expression of the Ion Extruders/Transporters	Inhibitors	Targeted Biological Function of Clinical Outcome	Biological Samples and/or Cell Lines Used	Refs.
Ewing Sarcoma	V_0_c, V_1_B_2_, and V_0_a_1_ V-ATPase	Bafilomycin A1, omeprazole, V_0_c V-ATPase siRNA	Cell viability and growth	A-673, SK-N-MC, RD-ES, SK-ES-1	[16]
Chondrosarcoma, osteosarcoma	V_0_c, V_1_B_2,_ and V_0_a_1_ V-ATPase	Esomeprazole alone or combined with sulphasalazine, omeprazole	Cell viability and motility, chemoresistance to doxorubicin, in vivo tumour growth, stemness	Primary cell cultures obtained from tumour biopsies, and Saos-2, SW1353, MG63, HOS, 143B, and RD cells and, 143B-mouse xenograft, frozen samples from human sarcoma and 3-methylcholanthrene (3-MCA)-induced sarcoma model.	[34,35,37,38]

**Table 2:** CA expression and targeting in bone sarcomas.

Types of Cancers	Expression of the Ion Extruders	Inhibitors Used	CA-Related Studied Biological Function or Clinical Outcome	Biological Samples and/or Cell Lines Used	Refs.
Chondrosarcoma, osteosarcoma	CAII and CAIX	CAIX, sulphonamide-derived inhibitors (and anti-HIF-1α inhibitors)	Cell viability, proliferation and motility, chemoresistance to doxorubicin, in vivo tumour growth, stemness	Primary cell cultures obtained from tumour biopsies, and Saos-2, SW1353, and MG63, HOS cells.	[34,39,40,41]
Osteosarcoma	CAVIII	None	Drug resistance, cell invasion, tumour growth, aerobic glycolysis	143b, HOS, MG63, U2-OS cells, and 143b xenografts	[42]
Fibrosarcoma	CAIX	None specific to CAIX (only HIF-1α inhibitors)	Hypoxia-modulated survival w/o and after irradiation	HT 1080 human fibrosarcoma cells and xenograft	[43,44]
Chondrosarcoma	CAIX	None	metastasis-free survival of patients	tumour biopsies	[45]

**Table 3:** MCT expression and targeting in bone sarcomas.

Types of Cancers	Expression of the Ion Extruders	Inhibitors Used	MCT-Related Studied Biological Function or Clinical Outcome	Biological Samples and/or Cell Lines Used	Refs.
Chondrosarcoma, osteosarcoma	MCT1	[alpha]-Cyano-4-hydroxycinnamate (CHC), shRNA anti MCT1	Cell viability and motility, chemoresistance to doxorubicin, in vivo tumour growth, stemness	Primary cell cultures obtained from tumour biopsies, and Saos-2, SW1353, MG63, MNNG/HOS, HOS, and 143B cells, and xenograft	[34,46,47]
Osteosarcoma	MCT4	none	Overall survival	Tumour biopsies	[48]

**Table 4:** NHE expression and targeting in bone sarcomas.

Types of Cancers	Expression of the Ion Extruders	Inhibitors Used	Targeted Biological Function	Biological Samples and/or Cell Lines Used	Refs.
Chondrosarcoma, osteosarcoma	NHE1	None	Cell viability and motility, chemoresistance to doxorubicin, in vivo tumour growth, stemness	Primary cell cultures obtained from tumour biopsies, and Saos-2, SW1353, and MG63, and HOS	[34]
